# Determinants of falls after stroke based on data on 5065 patients from the Swedish Väststroke and Riksstroke Registers

**DOI:** 10.1038/s41598-021-03375-9

**Published:** 2021-12-15

**Authors:** Carina U. Persson, Per-Olof Hansson

**Affiliations:** 1 Department of Clinical Neuroscience, Rehabilitation Medicine, Institute of Neuroscience and Physiology, Sahlgrenska Academy, University of Gothenburg, Per Dubbsgatan 14, 3rd Fl, Gothenburg, Sweden; 2 Department of Occupational Therapy and Physiotherapy, Sahlgrenska University Hospital/Östra, Gothenburg, Region Västra Götaland Sweden; 3 Department of Molecular and Clinical Medicine, Institute of Medicine, Sahlgrenska Academy, University of Gothenburg, Gothenburg, Sweden; 4 Department of Medicine, Geriatrics and Emergency Medicine, Sahlgrenska University Hospital/Östra, Gothenburg, Region Västra Götaland Sweden

**Keywords:** Health care, Neurology, Risk factors

## Abstract

We aimed to identify determinants in acute stroke that are associated with falls during the stroke unit stay. In order to enable individualized preventive actions, this knowledge is fundamental. Based on local and national quality register data on an unselected sample of 5065 stroke patients admitted to a stroke unit at a Swedish university hospital, univariable and multivariable logistic regression analyses were performed. The dependent variable was any fall during stroke unit stay. The independent variables related to function, activity, personal factors, time to assessment, comorbidities and treatments. Determinants of falls were: being male (odds ratio (OR) 2.25, 95% confidence interval (95% CI) 1.79–2.84), haemorrhagic stroke (OR 1.39, 95% CI 1.05–1.86), moderate stroke symptoms according to the National Institutes of Health Stroke Scale (NIHSS score 2–5 vs. NIHSS score 0–1) (OR 1.43, 95% CI 1.08–1.90), smoking (OR 1.70, 95% CI 1.29–2.25), impaired postural control in walking (OR 4.61, 95% CI 3.29–6.46), impaired postural control in standing (OR 1.60, 95% CI 1.25–2.05), stroke-related arm- and hand problems, OR 1.45, 95% CI 1.11–1.91), impaired cognition (OR 1.43, 95% CI 1.04–1.95), and urinary tract infection (OR 1.91, 95% CI 1.43–2.56). The findings from this study are useful in clinical practice and might help to improve patient safety after stroke.

## Introduction

After a stroke, one common and potentially dangerous consequence is a fall^1–3^. For the individual, the consequences of a fall may be not only a physical injury but also increasing dependence^4^, fear of falling^4^, impaired physical activity level^5^ and depressed mood^6^. It is therefore extremely important to minimize the risk of falling during hospital stays. Falls often occur early after admission to a stroke unit^7^. Consequently, in patient safety work, the early identification of individuals at risk of falling is essential. From a preventive point of view, in order to assist in the choice of the targeted implementation of effective preventive action, this identification is crucial.

Determinants of falls have been assessed at different time points after stroke onset^1,3,7–14^, but the number of studies that are based on assessments during the acute phase after a stroke, i.e. the first week after stroke onset, and related to falls during the inpatient rehabilitation is scare^7,15–17^. A small retrospective cohort study based on 113 individuals with acute stroke, showed that motor function in lower extremity movements associate with falls^15^. Cox et al. have performed a large retrospective study, including hospital stroke register data based on 856 patients suffering an ischemic stroke, which revealed that being male, previous myocardial infarction or renal insufficiency were the strongest predictors of falls^16^. In a large study based on 1809 patients with acute stroke, stroke severity, impaired spatial orientation and aphasia associated with falls^17^. Another study with a fairly large population, the Fall Study of Gothenburg (FallsGOT)^7^, which included both ischemic and haemorrhagic strokes, focused on assessments in acute stroke and identified determinants of falls during stroke unit stay as being male sex, poor or moderate postural control and the use of a walking aid in the acute phase after a stroke. In studies with limited study populations there is a risk of type 2 error, and a larger study population might identify additional determinants of falls. In addition, in FallsGOT, some selection occurred, as patients thought to be in need of thrombolysis or thrombectomy were referred to a stroke unit other than the study center and were not included. In patient safety work, the early identification of individuals at risk of falling is crucial. However, large-scale studies of acute stroke with unselected patient samples are scarce.

To address this knowledge gap, which implies few large and unselected stroke populations with examination of potential determinants of falls performed early after stroke, the aim of this study was to identify which factors prior to and shortly after a stroke are associated with falls during the stroke unit stay in a large, unselected sample of patients with a stroke. Based on previous studies and clinical experience, we hypothesized that high age, male sex, diabetes, impaired postural control, fall prior to the stroke, stroke severity and impaired cognition were all associated with falls.

## Methods

### Study design

This is a retrospective cohort study based on data from the local stroke register in Gothenburg, Väststroke, which includes patients with stroke and TIA admitted to any of three stroke units at Sahlgrenska University hospital^18^, and the National Swedish Stroke Register (Riksstroke)^19^. As Väststroke and Riksstroke complement one another by registering different variables, Väststroke data were linked to Riksstroke data. This link was made possible using the unique national 12-digit personal identity number used in Sweden. Ethical approval was obtained by the Swedish Ethical Review Authority (No.: 2019-02877). According to the Patient Data Act in Sweden, the processing of personal data in regional and national quality registers is permitted even without the written consent of the data subject. Need of informed consent was therefore waived by the Swedish Ethical Review Authority (Ethics Committee).

### Inclusion and exclusion criteria

The inclusion criteria were acute admission to any of the three stroke units at Sahlgrenska University hospital (SU), at Mölndal, Sahlgrenska or Östra, in Gothenburg, Sweden, during the period January 1, 2013 to October 15, 2019, with a diagnosis of an ischemic or haemorrhagic stroke (ICD-10 codes: I61, I63 and I64.9), admission directly to a stroke unit (not passing any other ward in hospital before arriving at the stroke unit) and with registered data in Väststroke on whether or not there was a fall during the stroke unit stay. A stroke unit is characterized by multidisciplinary teams with expertise related to stroke, with the emphasis on early mobilization and rehabilitation, detailed information on and the education of stroke victims and their relatives and programs for interventions, as well as regular follow-ups and quality assurance. Quality monitoring and improvement can be performed using data from quality registers.

The exclusion criteria were subarachnoid haemorrhage and any diagnosis other than stroke. In the case of more than one registered stroke event during the time period, only the first registration was used in the analyses.

### Assessment of the dependent variable

The dependent variable was at least one fall during the stroke unit stay. Falls data were collected from the Väststroke Register, in which the nurses at the stroke units registered whether the patients have experienced a fall/falls during the stroke unit stay or not (yes/no). A fall was defined as an event which results in a person coming to rest inadvertently on the ground or floor or other lower level^20^, regardless of whether or not an injury occurs.

### Assessment of the independent variables using data from Riksstroke and Väststroke registers

Baseline data were collected after admission to and during the stroke unit stay and were registered in the two registers, Riksstroke and Väststroke, during the stroke unit stay or shortly thereafter.

From Väststroke, we collected data on the following variables, potentially associated with falls: age; sex; type of stroke; stroke localization; stroke severity (using the National Institutes of Health Stroke Scale [NIHSS])^21^; postural control in sitting, standing and walking (the patient’s activity capacity for each of the three activities was classified as independent/dependent); previous fall/s; stroke-related arm and hand problems and cognition (by using the Montreal Cognitive Assessment [MoCA]^22^. The MoCA is a pencil-paper 30-point clinical test that assesses several cognitive domains. An MoCA score of < 26 was regarded as impaired cognition); previous physical activity level (using the Saltin Grimby Physical Activity Level Scale [SGPALS]. The SGPALS is a four-level scale ranging from 1 to 4, where a higher score refers to a higher physical activity level^23^); urinary tract infection during hospitalization and urinary catheterization. These data from Väststroke were merged with the following additional data, potentially associated with falls, collected from Riksstroke: time to first assessment by a physical therapist; diabetes mellitus; hypertension; atrial fibrillation; smoking and length of hospital stay.

### Statistical methods

Numbers and percentages were given for categorical data, while continuous and ordinal variables were specified by means, SD, medians, Q1, Q3 and min–max values. To identify associations between each independent variable and falls, univariable and multivariable logistic regression analyses were performed. The main statistical analysis was the logistic regression based on imputed values. Data for the main analysis were imputed using the stochastic imputation of all tentative determinants. For each variable, variables correlating with the imputation variable or the dichotomous missing/not missing pattern were included in the imputation, which was performed with regression or predictive mean matching using PROC MI (multiple imputation) in SAS with a predefined seed. Model selection was performed using best subset selection, choosing the model with the lowest mean Akaike information criterion over the 25 imputed datasets over all models.

Two sensitivity analyses were performed, one logistic regression using multiple imputation and one logistic regression on all available data. For the non-imputed multivariable model, variables significant at a p-value of ≤ 0.10 in the univariable analysis were entered in the forward logistic multivariable regression analysis. The unadjusted and the adjusted odds ratios with 95% confidence intervals (CIs) and p-values are presented. The area under the receiver operating characteristic (ROC) curve was calculated to describe the goodness of the model. All significance tests were two-sided and conducted at the 5% significance level.

## Results

**Figure 1:**
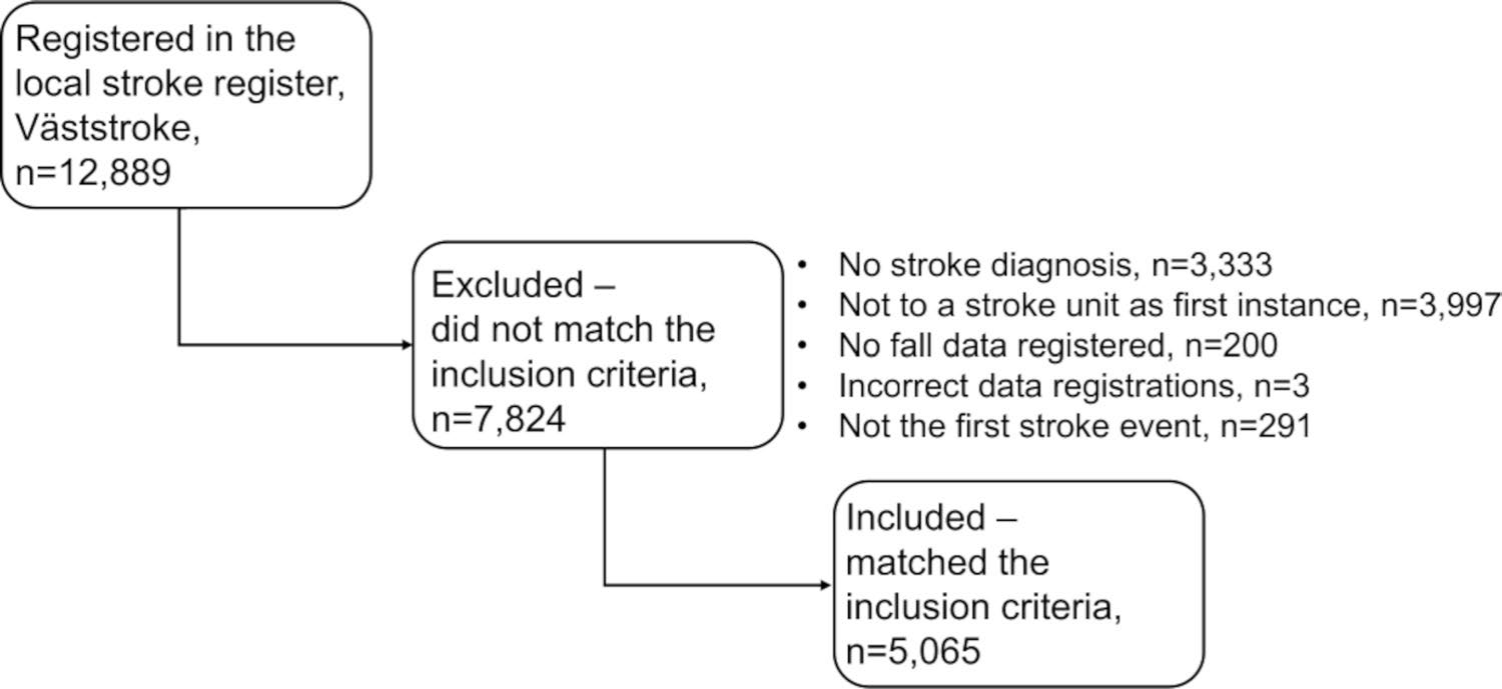
Flow chart for inclusion.

Of 12,889 registrations, 5065 patients met the inclusion criteria. Of these, 428 (8.5%) had experienced a fall. The flow chart of the inclusion is shown in Fig. 1. Table 1 presents original data compared with imputed data relating to descriptive statistics on the patients’ characteristics at baseline. The largest number of missing values was found for the NIHSS. Just under half were women and the median length of stay was just over 1 week. Fallers were older, mainly men and reported more frequently that they had been physically inactive prior to the stroke.

**Table 1:** Baseline characteristics, type of stroke and comorbidity among 5065 patients with acute stroke.

Characteristic	Original/imputed					
	Original			Imputed		
	Total	Non-fallers	Fallers	Total	Non-fallers	Fallers
		n = 4637	n = 428		n = 4637	n = 428
Age (years), median (min–max) (IQR)	76 (20; 104) (67; 85)	76 (20; 104) (67; 85)	78 (32; 100) (70; 85)	76 (20; 104) (67; 85)	76 (20; 104) (67; 85)	78 (32; 100) (70; 85)
Women	2397 (47.3%)	2243 (48.4%)	154 (36.0%)	2397 (47.3%)	2243 (48.4%)	154 (36.0%)
**Type of stroke 490**^**a**^**, 490**^**b**^						
Haemorrhagic	534 (11.7%)	459 (11.0%)	75 (19.3%)	534 (11.7%)	459 (11.0%)	75 (19.3%)
Ischemic	4041 (88.3%)	3727 (89.0%)	314 (80.7%)	4041 (88.3%)	3727 (89.0%)	314 (80.7%)
Stroke severity (NIHSS total score) 2078^a^	4.2 (5.7)	4.0 (5.6)	6.7 (6.5)	5.2 (6.7)	5.1(6.6)	6.8 (6.8)
Mean (SD)	2 (0; 32)	2 (0; 32)	4 (0; 24)	2 (0; 32)	2 (0; 32)	4 (0; 30)
Median (min–max) (IQR)	(0; 6)	(0; 5)	(2; 10)	(0; 8)	(0; 7)	(1; 10)
Thrombolysis treatment 44^a^	582 (11.6%)	539 (11.7%)	43 (10.1%)	588 (11.6%)	545 (11.8%)	43 (10.0%)
Receiving thrombectomy 392^a^	256 (5.5%)	217 (5.0%)	39 (10.4%)	299 (5.9%)	255 (5.5%)	44 (10.3%)
**Time to first assessment by a PT (min)**						
≤ 24 h	4036 (79.7%)	3690 (79.6%)	346 (80.8%)	4098 (80.9%)	3750 (80.9%)	348 (81.3%)
> 24 men ≤ 48 h	600 (11.8%)	538 (11.6%)	62 (14.5%)	608 (12.0%)	546 (11.8%)	62 (14.5%)
> 48 h	168 (3.3%)	152 (3.3%)	16 (3.7%)	169 (3.3%)	153 (3.3%)	16 (3.7%)
No assessment	186 (3.7%)	184 (4.0%)	2 (0.5%)	190 (3.8%)	188 (4.1%)	2 (0.5%)
Unknown if assessed or not	75 (1.5%)	73 (1.6%)	2 (0.5%)			
**Physical activity level (SGPALS) 1742**^**a**^						
(1) Physically inactive	1840 (55.4%)	1650 (54.4%)	190 (65.7%)	2848 (56.2%)	2570 (55.4%)	278 (65.0%)
(2) Some light physical activity	1290 (38.8%)	1205 (39.7%)	85 (29.4%)	1928 (38.1%)	1801 (38.8%)	127 (29.7%)
(3) Regular physical activity and training	186 (5.6%)	172 (5.7%)	14 (4.8%)	280 (5.5%)	257 (5.5%)	23 (5.4%)
(4) Regular hard physical training for competitive sports	7 (0.2%)	7 (0.2%)	0	9 (0.2%)	9 (0.2%)	0
**Comorbidities**						
Diabetes mellitus 39^a^	979 (19.5%)	878 (19.1%)	101 (23.7%)	991 (19.6%)	890 (19.2%)	101 (23.6%)
Hypertension 47^a^	2806 (55.9%)	2563 (55.8%)	243 (57.2%)	2836 (56.0%)	2591 (55.9%)	245 (57.2%)
Atrial fibrillation 37^a^	1377 (27.4%)	1240 (26.9%)	137 (32.2%)	1387 (27.4%)	1250 (27.0%)	137 (32.0%)
Smoking 606^a^	647 (14.5%)	581 (14.2%)	66 (17.7%)	745 (14.7%)	659 (14.2%)	86 (20.1%)
Postural control, independent, in walking 10 m indoors 1543^a^	1619 (46.0%)	1589 (49.4%)	30 (9.8%)	2221 (43.8%)	2162 (46.6%)	59 (13.8%)
Postural control, independent, in sitting 1858^a^	2714 (84.6%)	2500 (85.6%)	214 (74.3%)	4153 (82.0%)	3827 (82.5%)	326 (76.2%)
Postural control, independent, in standing 1989^a^	2234 (72.6%)	2124 (76.0%)	110 (39.1%)	3414 (67.4%)	3223 (69.5%)	191 (44.6%)
Previous fall 2431^a^	827 (31.4%)	730 (30.0%)	97 (48.7%)	1933 (38.2%)	1719 (37.1%)	214 (50.0%)
Stroke-related arm or hand problem 1832^a^	2197 (68.0%)	1959 (66.5%)	238 (82.9%)	3370 (66.5%)	3033 (65.4%)	337 (78.7%)
**Cognition (MoCA)—grouped**						
Normal capacity (MoCA score ≥ 26)	606 (12.0%)	587 (12.7%)	19 (4.4%)	1337 (26.4%)	1273 (27.5%)	64 (15.0%)
Impaired cognition (MoCA score < 26)	1232 (24.3%)	1096 (23.6%)	136 (31.8%)	3728 (73.6%)	3364 (72.5%)	364 (85.0%)
Not performed/unknown	3227 (63.7%)	2954 (63.7%)	273 (63.8%)			
Urinary catheter during hospital stay 394^a^	910 (19.5%)	780 (18.3%)	130 (32.3%)	998 (19.7%)	861 (18.6%)	137 (32.0%)
Urinary tract infection during hospital stay 83^a^	484 (9.7%)	407 (8.9%)	77 (18.5%)	506 (10.0%)	425 (9.2%)	81 (18.9%)
Length of stay (days) median (min–max) (IQR)	8 (1; 147) (4; 16)	7 (1; 147) (4; 14)	20 (2; 91) (13; 29)	8 (1; 147) (4; 16)	7 (1; 147) (4; 14)	20 (2; 91) (13; 29)

For categorical variables, n (%) is presented.

For continuous variables, the median (min; max) and (Q1; Q3) are presented.

There was no imputation for the 490 individuals who did not have data on the type of stroke.

*IQR* interquartile range, *NIHSS* the National Institute of Health Stroke Scale, *PT* physical therapist, *SGPALS* the Saltin-Grimby Physical Activity Scale, *MoCA* the Montreal Cognitive Assessment Scale.

^a^Indicates the number of missing data before the imputation of missing data.

^b^Indicates the number of missing data after the process of data imputation.

The results from the univariable and multivariable analyses on imputed values are presented in Table 2. In the univariable analysis, 17 of the independent variables were statistically significantly associated with falls, Table 2. These variables were all included in the multivariable analyses. Factors significantly associated with falls in the multivariable analysis were: being male, odds ratio (OR) 2.25 (95% confidence interval 1.79–2.84), having a haemorrhagic stroke, OR 1.39 (95% CI 1.05–1.86), having moderate stroke symptoms (NIHSS score 2–5 vs. NIHSS score 0–1): OR 1.43 (95% CI: 1.08–1.90), smoking, OR 1.70 (95% CI 1.29–2.25), impaired postural control (being dependent) when walking, OR 4.61 (95% CI 3.29–6.46), impaired postural control (being dependent) when standing, OR 1.60 (95% CI 1.25–2.05), stroke-related arm- and hand-related problems, OR 1.45 (95% CI 1.11–1.91), having impaired cognition, OR 1.43 (95% CI 1.04–1.95), and having a urinary tract infection, OR 1.91, (95% CI 1.43–2.56).

**Table 2:** Univariable and multivariable logistic regression for incidence of falling during hospital stay in 5065 patients with stroke (imputed data).

Variables	*	Value	n (%) of event	Univariable^a^			Multivariable^b^	
				OR (95% CI) dependent variable fall	p-value	Area under the ROC curve (95%CI)	OR (95% CI) dependent variable fall	p-value
Age (OR per 10 years)	0	20–< 71	119 (7.1%)					
		71–< 83	160 (9.1%)					
		83–104	149 (9.1%)	1.12 (1.04–1.21)	0.0043	0.54 (0.51–0.56)		
Sex	0	Woman (Ref.)	154 (6.4%)	1.00			1.00	
		Man	274 (10.3%)	1.67 (1.36–2.05)	< 0.0001	0.56 (0.54–0.59)	2.25 (1.79–2.84)	< 0.0001
Type of stroke	490	Infarction (Ref.)	314 (7.8%)	1.00			1.00	
		Haemorrhage vs. infarction	75 (14.0%)	1.94 (1.48–2.54)	< 0.0001	0.54 (0.52–0.56)	1.39 (1.05–1.86)	0.023
NIHSS score	0	0–1 (Ref.)	109 (5.2%)	1.00			1.00	
		2–5 vs 0–1	134 (9.8%)	1.96 (1.51–2.55)	< 0.0001		1.43 (1.08–1.90)	0.014
		6–32 vs 0–1	185 (11.4%)	2.33 (1.83–2.99)	< 0.0001	0.59 (0.57–0.62)	0.93 (0.70–1.25)	0.65
Time to physical therapist	261	Yes, ≤ 24 h (Ref.)	348 (8.5%)	1.00				
		Yes, > 24 but ≤ 48 h	62 (10.2%)	1.22 (0.92–1.63)	0.16			
		Yes, > 48 h	16 (9.5%)	1.13 (0.67–1.91)	0.66	0.51 (0.49–0.53)		
Diabetes mellitus	0	No (Ref.)	327 (8.0%)	1.00				
		Yes	101 (10.2%)	1.30 (1.03–1.64)	0.028	0.52 (0.50–0.54)		
Hypertension	0	No (Ref.)	183 (8.2%)	1.00				
		Yes	245 (8.6%)	1.06 (0.87–1.29)	0.59	0.51 (0.48–0.53)		
Atrial fibrillation	0	No (Ref.)	291 (7.9%)	1.00				
		Yes	137 (9.9%)	1.28 (1.03–1.58)	0.025	0.53 (0.50–0.55)		
Previous PA	0	(3 + 4) Regular physical activity and training or regular hard training for competitive sports (Ref.)	18 (5.9%)	1.00				
		(1) Physically inactive	283 (10.0%)	1.76 (1.08–2.88)	0.024			
		(2) Some light physical activity	127 (6.6%)	1.12 (0.67–1.86)	0.66	0.56 (0.53–0.58)		
Smoking	0	No (Ref.)	342 (7.9%)	1.00			1.00	
		Yes	86 (11.5%)	1.52 (1.18–1.95)	0.0011	0.53 (0.51–0.55)	1.70 (1.29–2.25)	0.0002
Postural control in walking	0	Independent (Ref.)	59 (2.7%)	1.00			1.00	
		Support/help	369 (13.0%)	5.46 (4.13–7.23)	< 0.0001	0.66 (0.65–0.68)	4.61 (3.29–6.46)	< 0.0001
Postural control in sitting	0	Independent (Ref.)	326 (7.8%)	1.00				
		Support/help	102 (11.2%)	1.48 (1.17–1.87)	0.0011	0.53 (0.51–0.55)		
Postural control in standing	0	Independent (Ref.)	191 (5.6%)	1.00			1.00	
		Support/help	237 (14.4%)	2.83 (2.31–3.46)	< 0.0001	0.62 (0.60–0.65)	1.60 (1.25–2.05)	0.0002
Previous fall	0	No (Ref.)	214 (6.8%)	1.00				
		Yes	214 (11.1%)	1.70 (1.39–2.07)	< 0.0001	0.56 (0.54–0.59)		
Arm and hand problem	0	No (Ref.)	91 (5.4%)	1.00			1.00	
		Yes	337 (10.0%)	1.96 (1.54–2.49)	< 0.0001	0.57 (0.55–0.59)	1.45 (1.11–1.91)	0.0069
Impaired cognition	0	Normal (Ref.)	64 (4.8%)	1.00			1.00	
		Impaired	364 (9.8%)	2.15 (1.64–2.83)	< 0.0001	0.56 (0.54–0.58)	1.43 (1.04–1.95)	0.027
MoCA (OR per 1 units)	0	2–< 18	201 (11.4%)					
		18–< 25	124 (7.8%)					
		25–30	103 (6.0%)	0.96 (0.95–0.97)	< 0.0001	0.60 (0.57–0.62)		
Urinary catheter	0	No (Ref.)	291 (7.2%)	1.00				
		Yes	137 (13.7%)	2.06 (1.66–2.56)	< 0.0001	0.57 (0.54–0.59)		
Urinary infection	0	No (Ref.)	347 (7.6%)	1.00				
		Yes	81 (16.0%)	2.31 (1.78–3.01)	< 0.0001	0.55 (0.53–0.57)	1.91 (1.43–2.56)	< 0.0001

There was no imputation for the 490 individuals who did not have data on the type of stroke.

Area under ROC curve with 95% CI for multivariable model = 0.76 (95% CI 0.73–0.78).

The Hosmer and Lemeshow test was non-significant (p = 0.50).

OR indicates odds ratio; 95% CI: 95% confidence interval; ROC: receiver operating characteristic; previous physical activity category 3 + 4 indicates regular physical activity and training or regular hard physical training for competitive sports; category 2: some light physical activity; category 1: physical inactivity; MoCA: the Montreal Cognitive Assessment scale.

p-values, OR and Area under ROC curve are based on original values and not on stratified groups.

OR is the ratio for the odds of an increase in the predictor of one unit.

For categorical variable groups, each group is compared with the rest.

^a^All tests are performed with univariable logistic regression.

^b^Multivariable logistic regression models are based on 4575 individuals and include: postural control when walking 10 m indoors; sex; urinary infection; postural control in standing; smoking (one or more cig./day or quit last 3 months); NIHSS group; stroke-related arm and hand problem; type of stroke (grouped) and cognition (MoCA) (grouped).

*The number of missing data before the imputation of missing data.

**Figure 2:**
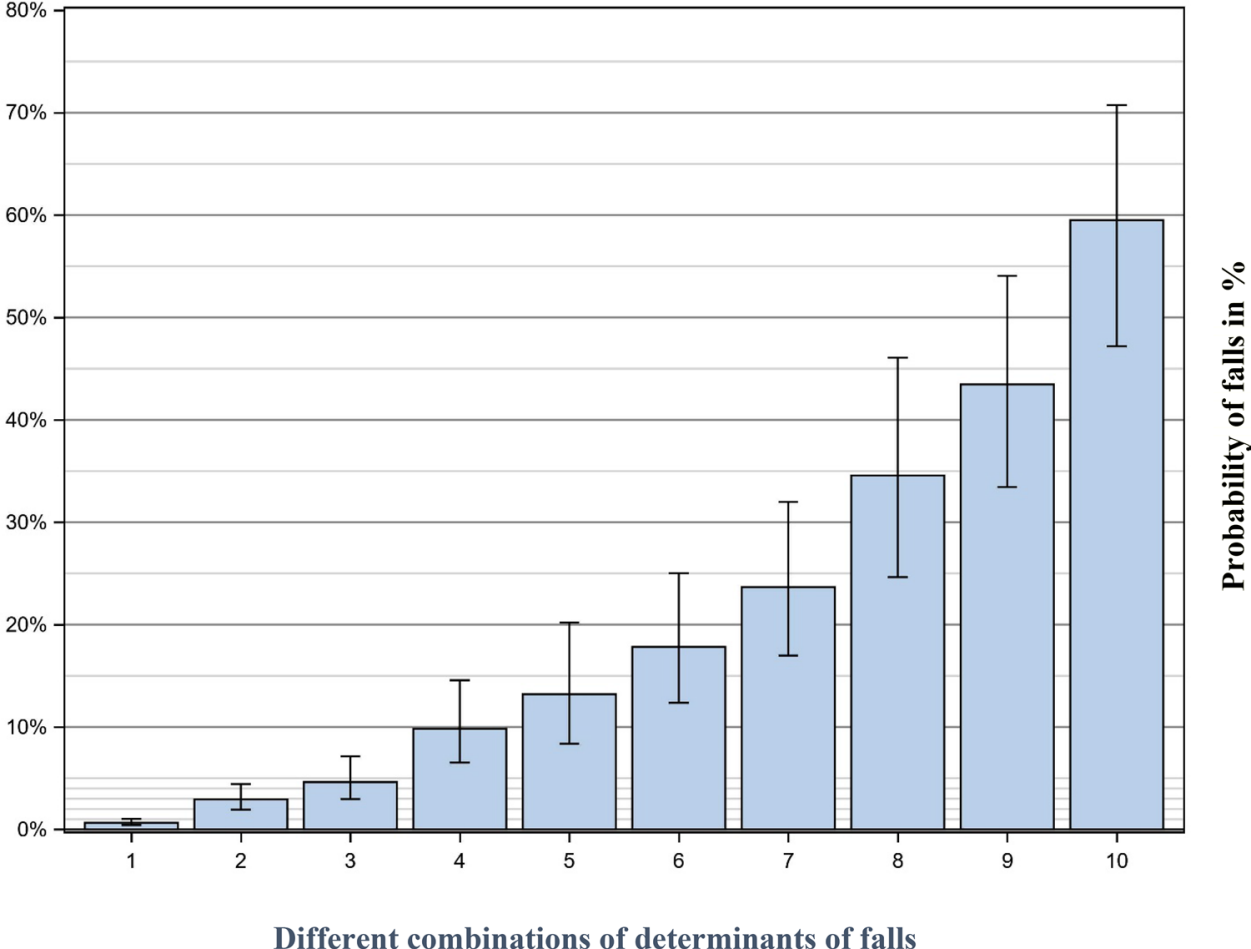
The probability of falls based on different combinations of significant determinants. (1) All significant determinants are at its most favourable, i.e.: good postural control in walking; good postural control in standing; woman; ischemic stroke; normal cognition NIHSS score 0–1; no smoking; normal arm-hand function; and no urinary tract infection (UTI). (2) Impaired postural control in walking, the other variables as in 1. (3) Impaired postural control in standing, the other variables as in 2. (4) Men, the other variables as in 3. (5) Haemorrhagic stroke, the other variables as in 4. (6) Impaired cognition, the other variables as in 5. (7) NIHSS score 2–5, the other variables as in 6. (8) Smoking, the other variables as in 7. (9) Impaired arm-hand function, the other variables as in 8. (10) UTI, the other variables as in 9.

Figure 2 shows the probability of falls based on the presence or absence of the determinants of falls. The probability of a fall is around 1% in patients with the most favourable outcome for all the determinants. As is shown, the risk of fall(s) increases with each additional determinant with the most negative outcome. For a patient with the most negative outcome for all determinants, the risk of fall(s) is just under 60%.

## Discussion

This study, based on register data from a sample composed of more than 5000 patients, is to this date the largest study on fall risk after stroke. We found that the determinants of falls during stroke unit stay are multifactorial. More specifically, the determinants are impaired postural control when standing and walking, being male, having a haemorrhagic stroke, having a stroke severity of 2–5 scores on the NIHSS, impaired cognition, smoking, urinary tract infection and stroke-related arm and hand problems. The strongest determinant is impaired postural control when walking. The fact that impaired postural control is strongly associated with falls post-stroke has previously been described and our study confirms this finding^7,10^. The multifactorial nature justifies an interdisciplinary approach in the early assessment of the patients admitted to the stroke unit. Our hypothesis regarding the association between fall/falls and male sex, stroke severity, having impaired postural control and impaired cognition was confirmed. However, the part of our hypothesis relating to high age, diabetes and a previous fall prior to the stroke was rejected. According to the non-significant Hosmer–Lemeshow test, the calibration was satisfactory. As a result, based on the area under the receiver operating characteristic curve, the prediction model showed an acceptable discrimination performance and is appropriate for use^24^.

Compared with the FallsGOT, the proportion of fallers was lower^7^. This was not surprising. In the FallsGOT, falls data were retrieved from both a web-based system for deviations and a review of medical records and this combination increased the number of identified falls. A previous large register study, based on patients with ischemic strokes and an average age of around 65 years, reported an even smaller proportion of fallers of 2.1%^16^. Also the study by Sinanovic et al. reported a low proportion (3.3%) of fallers^17^. When using only register data, as in the current study, there may be a risk of under-reporting the number of falls.

Age is associated with falls in the univariable analysis but not in the multivariable analysis. This is in line with the results from the FallsGOT^7^. As in other studies^7,16^, male sex is associated with falls post-stroke. The fact that almost two thirds of the fallers were men is in line with another register study of ischemic strokes, where 75% of the fallers were men^16^. In the current study, the odds ratio for a man falling was more than twice as high compared with female patients. The reason for this is not obvious and needs to be studied in more detail. Nevertheless, the finding is of clinical importance in a risk assessment at a stroke unit.

Patients suffering a haemorrhagic stroke are associated with falls. To the best of our knowledge, this interesting finding has not previously been described. The reason for this association is not obvious, but it might be related to the severe fatigue often occurring during the first weeks after an intracerebral haemorrhage. This is, however, only speculation.

In line with previous research^17^, patients with an NIHSS score of 2–5 ran a higher risk of falling compared with those with an NIHSS score of 0–1, while no significant difference was found for those with an NIHSS score of 6 or higher. Probably those with the highest NIHSS score were more immobilized and not exposed to circumstances in which they could experience a fall, at least in the acute phase after a stroke at the stroke unit. However, these patients with a more severe stroke still run a high risk of falling, during and after hospitalization. A previous study, with a 12-month follow-up, found that as many as 78% of those unable to walk 10 m in the acute phase after a stroke fell within the first year^1^.

We also found that smoking was an independent determinant of falling, in the multivariable analysis. This is another factor not previously described as a determinant of falls, where the reason is unclear. The patients are not allowed to smoke on the ward and anxiety and other abstinence symptoms might contribute to falls. In clinical practice, patients who smoke should be offered a transdermal nicotine patch. There are no data available relating to whether or not the patients were offered or prescribed a nicotine patch during the stroke unit stay.

The association between cognition and falls is consistent with a previous report, based on the subacute phase after stroke^12^. Patients with poor cognition may be less likely to be aware of a neurological deficit or impaired postural control, which could probably explain this increased risk of falling.

That a urinary tract infection was independently associated with falls is in line with previously research, where a urinary tract infection was associated with poor stroke outcome^25^. It is well known that all infections might affect both cognition and lead to fatigue among elderly persons. A urinary tract infection also causes an urge to urinate and being in a hurry to the toilet could cause a fall.

The current study is unique in terms of size, as it has a study population that is considerably larger than previous studies of determinants of falls in acute stroke^7,16^. By using data from two large registers of stroke patients, it was possible to address the problem of selection, enabling a very large, unselected population of patients with acute stroke and thereby complementing the literature by obtaining new knowledge of the way different factors are associated with falls in acute stroke.

The results of the study indicate that several factors contribute to the risk of falling in the acute phase after a stroke. Although the determinants of falls are multifactorial, they have the potential to be easily assessed and identified at no/low cost by the stroke team around the patient in their daily work, which might have important implications in clinical patient safety work. Physical therapists and occupational therapists play central roles in assessing postural control, walking and stroke-related arm and hand problems and cognition. Nurses, doctors, and every other member of staff can easily detect a urinary infection and make a note of sex and the type of stroke.

The main strength of the study is its large study population, making it possible to study a large variety of potential determinants in the acute phase after a stroke. The large sample size also makes it possible to detect factors with weaker associations. Another strength is the fact that we have an unselected stroke population and cover the vast majority of stroke patients in one geographic area, making the result representative and generalizable, at least for stroke patients in a western community. In addition, we adopted and used an accepted falls definition, which would enable comparisons between studies.

There are a number of clear limitations in this study. Primarily, being a register study, some factors that might relate to the risk of falling could not be studied, as data were not available. One such example is the use of a walking aid that, in previous research findings, identified as being associated with fall/s in acute stroke^7^ and after discharge from a stroke unit, regardless of ischemic or haemorrhagic strokes^3^. Another such example of factors not available in the two registers used in the current study is previous myocardial infection and renal insufficiency, which, in a large study of ischemic strokes, were the strongest predictors of falls, with odds ratios of 2.5 (95% CI 1.0–6.3) and 4.2 (95% CI 1.5–12.2) respectively. Moreover, in register studies, like the current study, the quality of data may vary, and a predictive relationship is not the same as a causal relationship. Further, data collection was performed according to real-life-settings at three different stroke units with many different team members. On the one hand, a limitation like this could be a variation in the scoring or choice of criteria between all the team members. On the other hand, one advantage of a real-life-setting could be that it is ideal for describing a standard and as such strengthens the ecological validity. As a result, the fact that all the determinants have been acquired in everyday clinical work facilitates the potential to comply with these research findings in clinical work. In addition to the comment related to generalizability above, our study findings cannot be generalized to apply to patients other than those with an acute and subacute stroke. Finally, there were initially a fairly large number of missing values, especially related to cognition (MoCA). However, in order to minimize the impact of this, multiple imputation was used in the statistical analysis. Further research should identify the value of implementing the newly acquired knowledge in clinical practice, on how to identify individuals at risk of falling and interventions to influence modifiable factors.

## Conclusions

Falls during stroke unit stay are multifactorial by nature and justifies an interdisciplinary approach. Nine factors were identified as determinants, having them all constitutes a 59% probability of falling during stroke unit stay. The strongest determinant of falls was impaired postural control when walking. The findings in this study are useful in clinical practice in order to identify patients with increased risk of falling, who might benefit from increased attention.
